# 7 versus 14 days of antibiotic treatment for critically ill patients with bloodstream infection: a pilot randomized clinical trial

**DOI:** 10.1186/s13063-018-2474-1

**Published:** 2018-02-17

**Authors:** Nick Daneman, Asgar H. Rishu, Ruxandra Pinto, Pierre Aslanian, Sean M. Bagshaw, Alex Carignan, Emmanuel Charbonney, Bryan Coburn, Deborah J. Cook, Michael E. Detsky, Peter Dodek, Richard Hall, Anand Kumar, Francois Lamontagne, Francois Lauzier, John C. Marshall, Claudio M. Martin, Lauralyn McIntyre, John Muscedere, Steven Reynolds, Wendy Sligl, Henry T. Stelfox, M. Elizabeth Wilcox, Robert A. Fowler, on behalf of the Canadian Critical Care Trials Group

**Affiliations:** 1 Division of Infectious Diseases and Clinical Epidemiology, Sunnybrook Health Sciences Centre, University of Toronto and Adjunct Scientist, Institute for Clinical Evaluative Sciences, Sunnybrook Health Sciences Centre, 2075 Bayview Ave, Toronto, ON M4N 3M5 Canada; 2 Department of Critical Care Medicine, Sunnybrook Health Sciences Center, Toronto, ON Canada; 3 Service de Soins Intensifs et Centre de Recherche, Centre Hospitalier de l’Université de Montréal, Montréal, QC Canada; 4 Department of Critical Care Medicine, Faculty of Medicine and Dentistry, University of Alberta, Edmonton, AB Canada; 5 Department of Microbiology and Infectious Diseases, Université de Sherbrooke, Sherbrooke, QC Canada; 6 Department of Critical Care Medicine, Hôpital du Sacré-Coeur de Montreal and Hôpital de Trois-Rivières, University of Montreal, Montreal, QC Canada; 7 Division of Infectious Diseases, University of Toronto, Toronto, ON Canada; 8 Division of Critical Care Medicine, Department of Medicine, McMaster University, Hamilton, ON Canada; 9 Division of Critical Care, Department of Medicine, Sinai Health System, Toronto, ON Canada; 10 Division of Critical Care Medicine and Center for Health Evaluation and Outcome Sciences, St. Paul’s Hospital and University of BC, Vancouver, BC Canada; 11 Departments of Critical Care Medicine and Anesthesiology, Pain Management and Perioperative Medicine, Dalhousie University, Halifax, NS Canada; 12 Section of Critical Care Medicine, University of Manitoba, Winnipeg, MB Canada; 13 Centre de Recherche du CHU de Sherbrooke and Department of Medicine, Université de Sherbrooke, Sherbrooke, QC Canada; 14 Centre de Recherche du CHU de Québec-Université Laval, Axe Santé des Populations et Pratiques Optimales en Santé, Division de Soins Intensifs, Québec, QC Canada; 15 Departments of Surgery and Critical Care Medicine, St. Michael’s Hospital, University of Toronto, Toronto, ON Canada; 16 Department of Medicine, University of Western Ontario, London, ON Canada; 17 Division of Critical Care, Department of Medicine, The Ottawa Hospital, Ottawa, ON Canada; 18 Department of Critical Care Medicine, Queen’s University, Kingston, ON Canada; 19 Department of Biophysiology and Kinesiology, Simon Fraser University, Burnaby, BC Canada; 20 Department of Critical Care Medicine, Institute of Public Health, University of Calgary, Calgary, AB Canada; 21 Division of Critical Care, Department of Medicine, Toronto Western Hospital, Toronto, ON Canada; 22 Departments of Medicine and Critical Care Medicine, Sunnybrook Health Sciences Center, Adjunct Scientist, Institute for Clinical Evaluative Sciences, Sunnybrook Health Sciences Centre, Institute of Health Policy, Management and Evaluation, University of Toronto, Toronto, ON Canada

**Keywords:** Bacteremia, Bloodstream infection, Critical care, Intensive care, Duration of treatment

## Abstract

**Background:**

Shorter-duration antibiotic treatment is sufficient for a range of bacterial infections, but has not been adequately studied for bloodstream infections. Our systematic review, survey, and observational study indicated equipoise for a trial of 7 versus 14 days of antibiotic treatment for bloodstream infections; a pilot randomized clinical trial (RCT) was a necessary next step to assess feasibility of a larger trial.

**Methods:**

We conducted an open, pilot RCT of antibiotic treatment duration among critically ill patients with bloodstream infection across 11 intensive care units (ICUs). Antibiotic selection, dosing and route were at the discretion of the treating team; patients were randomized 1:1 to intervention arms consisting of two fixed durations of treatment – 7 versus 14 days. We recruited adults with a positive blood culture yielding pathogenic bacteria identified while in ICU. We excluded patients with severe immunosuppression, foci of infection with an established requirement for prolonged treatment, single cultures with potential contaminants, or cultures yielding *Staphylococcus aureus* or fungi. The primary feasibility outcomes were recruitment rate and adherence to treatment duration protocol. Secondary outcomes included 90-day, ICU and hospital mortality, relapse of bacteremia, lengths of stay, mechanical ventilation and vasopressor duration, antibiotic-free days, *Clostridium difficile*, antibiotic adverse events, and secondary infection with antimicrobial-resistant organisms.

**Results:**

We successfully achieved our target sample size (*n* = 115) and average recruitment rate of 1 (interquartile range (IQR) 0.3–1.5) patient/ICU/month. Adherence to treatment duration was achieved in 89/115 (77%) patients. Adherence differed by underlying source of infection: 26/31 (84%) lung; 18/29 (62%) intra-abdominal; 20/26 (77%) urinary tract; 8/9 (89%) vascular-catheter; 4/4 (100%) skin/soft tissue; 2/4 (50%) other; and 11/12 (92%) unknown sources. Patients experienced a median (IQR) 14 (8–17) antibiotic-free days (of the 28 days after blood culture collection). Antimicrobial-related adverse events included hepatitis in 1 (1%) patient, *Clostridium difficile* infection in 4 (4%), and secondary infection with highly resistant microorganisms in 10 (9%). Ascertainment was complete for all study outcomes in ICU, in hospital and at 90 days.

**Conclusion:**

It is feasible to conduct a RCT to determine whether 7 versus 14 days of antibiotic treatment is associated with comparable 90-day survival.

**Trial registration:**

ClinicalTrials.gov, identifier: NCT02261506. Registered on 26 September 2014.

**Electronic supplementary material:**

The online version of this article (10.1186/s13063-018-2474-1) contains supplementary material, which is available to authorized users.

## Background

Both antibiotic use and the acquisition of antibiotic-resistant organisms are high in intensive care units (ICUs), where critically ill patients are vulnerable to bacterial infections and antibiotic complications. Shortened treatment durations offer an appealing opportunity to maximize the benefits while minimizing the harms and reducing the costs of antibiotic therapy [1]. Randomized clinical trials (RCTs) have established that shorter-duration treatments are sufficient for a wide range of bacterial infections [1–5], including some infections among critically ill patients [6], but the optimal treatment duration for bloodstream infections remains understudied.

Our systematic review of the academic literature revealed no trials of shorter- versus longer-duration treatment among adult patients with bloodstream infection, but did uncover 24 studies (of 7595 patients) of shorter versus longer antibiotic treatment for infections commonly complicated by bacteremia. Cure rates were similar in patients receiving shorter (3–7 days) versus longer (7–21 days) treatment (risk ratio 1.00, 95% confidence interval (CI) 0.98–1.01) [7]. Although, subgroup outcome data were only available for small numbers of bacteremic patients across these studies (*n* = 115), shorter-duration treatment was associated with similar clinical cure (45/52 versus 47/49, risk ratio 0.88, 95% CI 0.77–1.01) and survival rates (15/17 versus 26/29, risk ratio 0.97, 95% CI 0.76–1.23) as longer-duration treatments. Given the absence of evidence to guide treatment durations for these bacteremic patients, we conducted a national practice survey of infectious disease physicians and critical care physicians [8]. Among these clinicians, 14 days was the most commonly reported treatment duration, but shorter treatments (usually 7 or 10 days) were recommended by nearly half of practitioners [8]. In single [9] and multicenter [10] observational studies of actual practice, we confirmed that critically ill patients with bacteremia receive a median of 14 days of treatment, but with wide variability (interquartile range (IQR) 9–17.5 days), further justifying the need for a trial comparing 7 versus 14 days of treatment. If such a trial could establish that 7 days is non-inferior to 14 days of treatment, this could lead to massive reductions in antimicrobial use, resistance and complications, and an estimated annual cost savings of $678 to 798 million in North America and $1.4 to 1.6 billion across Europe [11].

Prior to embarking on a large multicentre trial, we conducted a pilot RCT to test the feasibility of this trial design. We hypothesized that the co-primary feasibility outcomes (recruitment rate and adherence to treatment duration protocol) would confirm that a definitive trial could be conducted to compare 7 versus 14 days of antibiotic treatment for critically ill patients with bloodstream infections.

## Methods

### General study design

We conducted an open, pilot RCT of 7 versus 14 days of antibiotic treatment, for critically ill patients with bloodstream infection, to test the feasibility and inform the design of an international, non-inferiority RCT. The trial protocol of the Bacteremia Antibiotic Length Actually Needed for Clinical Effectiveness (BALANCE) pilot RCT has been previously published [12]; however, the key design elements are summarized below. We have adhered to the Consolidated Standards of Reporting Trials (CONSORT) guideline for pilot trials [13].

### Study setting

The study was conducted through the Canadian Critical Care Trials Group (CCCTG) at 11 participating ICUs, across 10 Canadian cities in five provinces. The ICUs started in staggered fashion (Sunnybrook Health Sciences Centre, Toronto, ON (October 2014); Kingston General Hospital, Kingston, ON (January 2015); Queen Elizabeth II, Halifax, NS (March 2015); The Ottawa Hospital, Ottawa, ON (June 2015); Université de Sherbrooke, Sherbrooke, Québec, QC (July 2015); St. Boniface Hospital, Winnipeg, MB (January 2016); L’Hôpital de l’Enfant-Jesus, Laval, Québec, QC (January 2016); CHUM, Montréal, Québec, QC (April 2016); Mount Sinai Hospital, Toronto, ON (April 2016); London Health Sciences Centre, London, ON (May 2016); and University of Alberta Hospital, Edmonton, AB (June 2016)). Patient enrollment was completed in July 2016, and thus individual ICUs participated for durations ranging from 1 to 22 months. Institutional Research Ethics Board approval was obtained at these participating sites.

### Inclusion criteria

The inclusion criteria were intended to be broad, so as to be generalizable to most critically ill patients with bloodstream infection. All adult patients (aged over 18 years) with any positive blood culture yielding a pathogenic bacterium while in the ICU were included. The blood culture need not have been collected in the ICU, but at the time of detection of the positive result the patient needed to be under ICU care.

### Exclusion criteria

We excluded patients who were severely immunocompromised (only neutropenia or bone marrow, solid organ or stem cell transplantation); had prosthetic valves or synthetic endovascular grafts; had a suspected or documented syndrome with an established requirement for prolonged treatment (e.g., infective endocarditis, osteomyelitis/septic arthritis, undrainable/undrained abscess or unremovable/unremoved prosthetic infection); had a single positive blood culture with coagulase-negative staphylococci, *Bacillus* spp., *Corynebacterium* spp., etc. (because these may have represented contamination rather than infection); had positive blood cultures with *Staphylococcus aureus* or fungal organisms (because observational data suggest that these warrant more prolonged treatment); or patients previously enrolled in this trial.

### Intervention: 7 versus 14 days of adequate antimicrobial treatment

Eligible consenting patients were randomized 1:1 to receive a shorter duration of adequate antimicrobial treatment (7 days) versus a longer duration (14 days). Adequate antimicrobial treatment was defined as a regimen with in vitro activity against the organism(s) responsible for the bloodstream infection. The duration of adequate treatment was determined as the cumulative number of calendar days for which adequate treatment was delivered including and beyond the date of collection of the index blood culture specimen [12]. The selection of antimicrobial agents, doses, intervals and routes of delivery were left to the discretion of the clinical team; only the duration was determined by randomization.

### Randomization, blinding, and allocation concealment methods

Randomization was accomplished via a central, web-based system (http://www.randomize.net) using variable block sizes, stratified by ICU site; patients were allocated 1:1 into 7- versus 14-day treatment arms. Although placebo controls have been used in some RCTs of antibiotic treatment duration [3, 14–19], we believe that they are not practical or appropriate for bacteremia treatment in ICU because of variable pathogens, sources of bacteremia, mono- and combination antibiotic treatment regimens, and the potential for critically ill patients to develop secondary nosocomial infections requiring ongoing re-assessment of treatment choices. We elected not to protocolize treatment choices given the variety of pathogens and infectious syndromes causing the bacteremia, so as to maximize generalizability of study findings. As a consequence, patients and clinicians were not blinded to treatment assignment. To mitigate selection bias, and prevent clinicians from providing differential treatments to patients in the two arms, we maintained allocation concealment until the end of day 7, an approach used successfully in the *PneumoA* RCT of shorter- versus longer-duration antibiotic treatment for ventilator-associated pneumonia [6].

### Primary feasibility outcomes

The co-primary outcomes of this pilot RCT related to feasibility of the main trial: (1) recruitment rates and (2) adherence to assigned treatment duration protocol. We targeted overall recruitment rates of one patient per ICU site per month; we targeted protocol adherence rates of 90% of antibiotic treatment courses within 7 ± 2 days in the shorter-duration arm, and 14 ± 2 days in the longer-duration arm.

### Secondary clinical outcomes

Secondary outcomes included 90-day mortality, which is the planned primary outcome of the larger RCT, and the best measure of net treatment efficacy, as well as other measures of net efficacy including ICU and hospital mortality, relapse of bacteremia, ICU and hospital lengths of stay (LOS), mechanical ventilation and vasopressor duration. Other secondary outcomes include antibiotic-free days, as well as measures of antibiotic-related harms, including rates of *Clostridium difficile* infection, antibiotic adverse events (allergy, anaphylaxis, acute kidney injury, hepatitis) and secondary nosocomial infection with antimicrobial-resistant organisms. Antibiotic-free days were defined as the number of calendar days within 28 days after blood culture collection on which the patient did not receive any antibiotic treatments; any patient dying within 28 days of blood culture collection was assigned zero antibiotic-free days. A composite definition of highly resistant microorganisms was modified from the description by de Smet et al. [20] as previously described [21].

### Mechanistic sub-studies

Within the framework of this study, we also piloted two mechanistic sub-studies with plans for expansion within the larger RCT. First, in a procalcitonin (PCT) sub-study, we obtained blood PCT measurements on the day of randomization, and days 7, 10, and 14 from the index blood culture collection. The results were not made available to the treating team because this could have unduly influenced protocol adherence; rather, they were batch-tested at the end of the trial using VIDAS® B.R.A.H.M.S. PCT™, BioMérieux (Marcy L’Etoile, France). The outcome of interest was the proportion of patients for whom PCT levels exceeded the usual threshold (0.25 IU/mL) for recommending antibiotic treatment at day 7 and day 14. At a single ICU site, we also collected rectal swabs on the day of enrollment, day 7, day 14, and at either hospital discharge or 28 days post enrollment, for analysis of gut microbial diversity and bacterial community composition by *16S rRNA* gene sequencing, with the main outcome being taxonomic diversity (Shannon Diversity Index) [22].

### Data collection and follow-up details

Patients were followed daily during the hospital stay, with entry of baseline characteristics, and outcome information into a secure, electronic Case Report Form. The secondary outcome (90-day mortality) was collected by follow-up telephone call at 90 days from the index bacteremia.

### Statistical analysis

The analytic plan for the pilot RCT was primarily descriptive – determining the recruitment rate, overall and by ICU site, and calculating the protocol adherence rate overall, by ICU site, and by source of infection. A pilot RCT is not adequately powered to identify a clinically important difference in safety endpoints or in overall mortality among patients receiving shorter- versus longer-duration treatments – rather this is the intended goal of the BALANCE main RCT. Therefore, as per the approach of most pilot trials conducted by the CCCTG, we did not un-blind treatment assignment in the BALANCE pilot RCT [23]. Avoiding such underpowered analyses for clinical endpoints decreases the risk of over-interpreting such results, which could unduly influence investigators, clinicians, peer-reviewers, and research ethics boards [23].

### Sample size calculation

Our pilot RCT sample size (*n* = 115) was calculated such that we would be able to estimate our protocol adherence rate within a margin of error of ± 5%, with 95% confidence, with an adherence rate of 90%; or ± 7%, with 95% confidence, with an anticipated adherence rate of 80%.

## Results

### Screened, eligible and randomized patients

**Fig. 1:**
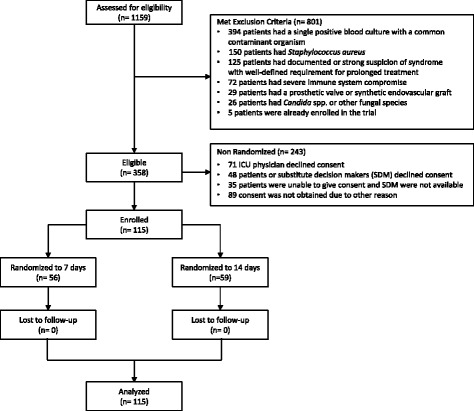
Consolidated Standards of Reporting Trials (CONSORT) flow diagram describing eligibility screening and randomization assessments

In total, 1159 critically ill patients with a positive blood culture were screened for eligibility, of whom 358 (31%) were eligible for enrollment (Fig. 1 CONSORT flow diagram; Additional file 1). Among 801 patients who met the exclusion criteria, the most common reasons for non-eligibility were single positive cultures with a common contaminant organism (49%), *Staphylococcus aureus* bacteremia (19%), suspected or documented syndrome with well-defined requirement for prolonged treatment (16%), severe immuncompromise (9%), prosthetic valve or synthetic endovascular graft (4%) or fungemia (3%). Of the eligible patients, 115/358 (32%) were randomized into the study; the most common reasons for non-randomization were lack of consent by the ICU physician (29%) or the patient or substitute decision-maker (SDM) (20%), or the lack of an available SDM (14%) (Fig. 1). There was no loss to follow-up and no missing outcome data.

### Patient, infection, and pathogen characteristics

Patients had a median age of 67 (IQR, 57–78) years and were severely ill with a median Acute Physiology And Chronic Health Evaluation (APACHE II) score of 22 (IQR, 18–26); 63 (55%) were male (Table 1). Most bloodstream infections were community-acquired (60%), with the remainder acquired on hospital wards (17%) or in the ICU (24%). The most common sources of bacteremia were lung (27%), abdomen (25%), and urinary tract (23%); the most common pathogens were *Escherichia coli* (22%), *Klebsiella* spp. (14%), and *Enterococcus* spp. (13%), but there was a total of 25 different bacteria isolated among the index blood cultures of these patients (Table 1).

**Table 1:** Patient, infection, and pathogen characteristics among critically ill patients with bloodstream infection

Characteristic^a^	*N* (%), Median (IQR)
Patient characteristic	
Male sex	63 (55)
Age in years	67 (57–78)
APACHE II Score	22 (18–26)
Baseline vasopressor use	60 (52)
Admission category	
Medical	78 (68)
Surgical	24 (21)
Trauma	6 (5)
Neurological/neurosurgical	6 (5)
Burns	1 (1)
Comorbidity	
Coronary artery disease	23 (20)
Congestive heart failure	16 (14)
Arrhythmia	15 (13)
Peripheral vascular disease	14 (12)
Diabetes mellitus	40 (35)
Renal insufficiency	13 (11)
Dialysis dependency	4 (4)
Chronic obstructive pulmonary disease	16 (14)
Liver disease	8 (7)
Obesity	16 (14)
Solid malignancy	18 (16)
Leukemia/lymphoma	1 (1)
Steroids/immunosuppression	10 (9)
Infection characteristics	
Acquisition of bacteremia	
Community-acquired	69 (60)
Hospital-acquired	19 (17)
ICU-acquired	27 (24)
Source of bacteremia	
Lung	31 (27)
Intra-abdominal/hepato-biliary	29 (25)
Urinary tract	26 (23)
Vascular-catheter-related	9 (8)
Skin and/or soft tissue	4 (3)
Other	4 (3)
Undefined/unknown	12 (10)
Top 10 most commonly isolated pathogens in blood cultures^b^	
*Escherichia coli*	28 (22)
*Klebsiella* spp.	18 (14)
*Enterococcus* spp.	17 (13)
*Streptococcus pneumoniae*	13 (10)
Coagulase-negative staphylococci	12 (9)
*Enterobacter* spp.	6 (5)
*Pseudomonas* spp.	4 (3)
*Serratia* spp.	4 (3)
*Citrobacter* spp.	3 (2)
*Streptococcus anginosus* group	3 (2)

^a^All data are presented as medians and interquartile ranges (IQR) unless otherwise specified

^b^A total of 24 different bacterial species were isolated among the index blood cultures of the 115 patients; the denominator for these percentages is all organisms isolated from patients’ index blood cultures

### Treatment characteristics

A total of 31 different antimicrobials were used in treating these patients, with the most common being piperacillin-tazobactam (in 73.9% of patients), vancomycin (56.5%), ceftriaxone (50.4%), ciprofloxacin (36.5%), and meropenem (30.4%) (Table 2). Patients received a median of 2 (IQR, 1–2) adequate antimicrobials in the empiric window prior to blood culture finalization, and 3 (IQR 2–3) in the period up to 30 days after blood culture collection. A source control procedure was required for 76/115 (66.1%) patients.

**Table 2:** Treatment characteristics among critically ill patients with bacteremia

Treatment characteristics^a^	*N* (%), Median (IQR)
Empiric antimicrobial treatment^b^	
Number of unique empiric antimicrobials administered	2 (2–3)
Number of unique adequate^d^ empiric antimicrobials administered	2 (1–2)
Overall antimicrobial treatments^c^	
Number of unique antimicrobials administered	3 (3–5)
Number of unique adequate^d^ antimicrobials administered	3 (2–3)
Top 10 most commonly administered antimicrobials	
Piperacillin-tazobactam	85 (73.9%)
Vancomycin	65 (56.5%)
Ceftriaxone	58 (50.4%)
Ciprofloxacin	42 (36.5%)
Meropenem	35 (30.4%)
Metronidazole	22 (19.1%)
Cefazolin	20 (17.4%)
Ampicillin	19 (16.5%)
Tobramycin	14 (12.2%)
Amoxicillin-clavulanate	11 (9.6%)
Underwent a source control procedure	76 (66.1%)

^a^All data are presented as medians and interquartile ranges (IQR) unless otherwise specified

^b^Empiric treatment window defined as period between blood culture collection and finalization

^c^Overall treatments received within 30 days after blood culture collection

^d^Adequate antimicrobials defined by in vitro activity against the blood culture pathogen(s)

### Recruitment rate

The overall average recruitment rate was one patient per site per month. Across individual sites the median (IQR) recruitment rate was 0.7 (0.3–1.5) patients per month, with a range of 0–2.1 patients per month.

### Adherence to treatment duration protocol

The overall adherence to assigned treatment duration protocol was 89/115 (77%). Across individual sites the median (IQR) adherence to treatment duration protocol was 71% (50–85%). Adherence to treatment duration varied according to underlying source of infection: 26/31 (84%) lung; 18/29 (62%) intra-abdominal; 20/26 (77%) urinary tract; 8/9 (89%) vascular-catheter; 4/4 (100%) skin and soft tissue; 2/4 (50%) other; and 11/12 (92%) unknown sources of infection. The most common reasons for protocol non-adherence were re-starting of antibiotics for documented/suspected recurrence or persistence of the index infection (6), clinical reasons for extended treatment that were not present or unrecognized at time of enrollment (4), a new infection unrelated to the index infection (4), ward physician not agreeing with ICU orders after transfer (2), recent surgery (1), and recent prosthetic valve insertion (1). In the 14-day treatment arm, the most common reasons for protocol non-adherence were clinical reasons for extended treatment that were not present or unrecognized at time of enrollment (2), a new infection unrelated to the index infection (2), physician error (1), new gastrointestinal tract perforation (1), life-sustaining treatment withdrawal (1), and recurrence or persistence of index infection (1). There was a trend towards higher protocol adherence among the second half of the patient group enrolled at each ICU site (82%) as compared to the first half of the enrolled patient group at each site (73%) (*p* = 0.24).

### Other feasibility outcomes

No patients were withdrawn from the trial, and there were no losses to follow-up, with complete ascertainment of all study outcomes in ICU, in hospital, and at 90 days.

### Clinical outcomes

The overall mortality rate among randomized patients was 7% in ICU, 13% in hospital, and 15% at 90 days (Table 3). Relapse of bacteremia with the same pathogen occurred in 4 (3%) patients. Patients experienced a median of 14 antibiotic-free days, with wide variability (IQR 8–17 days), and bimodal distribution (modes at 14 and 21 antibiotic-free days). No patients experienced allergy/anaphylaxis or acute kidney injury. There was one episode of acute hepatitis (1%), four patients (3%) with *Clostridium difficile* infection, and 10 (9%) with secondary infection with a highly resistant microorganism, including four with methicillin-resistant *Staphylococcus aureus*, two with vancomycin-resistant *Enterococci*, two with extended-spectrum beta-lactamase (ESBL)-producing *Enterobacteriaceae*, two with multi-drug resistant *Enterobacteriaceae* and one with multi-drug resistant non-*Enterobacteriaceae*.

**Table 3:** Clinical outcomes among critically ill patients with bacteremia

Outcome^a^	*N* (%), Median (IQR)
Mortality	
in ICU	8 (7)
in hospital	15 (13)
at 90 days	17 (15)
Length of stay (in days)	
in ICU	8 (4–20)
in hospital	20 (12–42)
Duration of life support (in days)	
mechanical ventilation	8 (3–21)
Relapse of bacteremia	4 (4)
Antibiotic-free days (by day 30)	14 (8–17)
Antimicrobial-related adverse outcomes	
Allergy	0 (0)
Anaphylaxis	0 (0)
Acute kidney injury	0 (0)
Acute hepatitis	1 (1)
*Clostridium difficile* infection	4 (4)
Secondary infection with highly resistant microorganisms	10 (9)

^a^All data are presented as medians and interquartile ranges (IQR) unless otherwise specified. *ICU* intensive care unit

### Mechanistic sub-studies

The majority of patients (101/115, 88%) consented to optional blood testing for procalcitonin (PCT) measurements. The median (IQR) PCT levels were 5.0 (2.0–22.5) IU/mL on the day of randomization, 1.3 (0.3–4.3) on day 7, 0.46 (0.15–1.55) on day 10, and 0.28 (0.10–1.19) on day 14. Only a minority of patients had PCT levels below the 0.25 threshold at which PCT algorithms would recommend antibiotic discontinuation, including 12/71 (17%) on the day 7 measurement, and 21/47 (45%) on the day-14 measurement. Most patients (27/37, 73%) at the central study site consented to rectal swabs for microbiome testing, which confirmed the adequacy of this sampling method for bacterial community testing, and considerable range in the Shannon Diversity Index, with a median (range) of 4.1 (0.2–5.5) across all specimens.

## Discussion

The BALANCE pilot RCT has confirmed the feasibility of conducting a large, multicenter RCT comparing 7 versus 14 days of antibiotic treatment for critically ill patients with bloodstream infection. It was feasible to recruit patients into this trial, and adherence to treatment duration protocols was adequate to ensure wide separation in antibiotic treatment between groups. No patients were withdrawn from the trial, and there were no losses to follow-up in ascertainment of any of the study outcomes in ICU, in hospital, or at 90 days.

Although, there was some heterogeneity in recruitment rates across sites, we achieved our overall target recruitment average of one patient per ICU per month. The principle BALANCE trial will seek to test whether 7 days of treatment is associated with a non-inferior survival rate as compared to 14 days of treatment, with a non-inferiority margin of 4%; after inflation for two interim analyses and potential losses to follow-up, the sample size requirement will be 3598. Therefore, at the rate of recruitment in our pilot RCT we will require approximately 60 ICUs, recruiting patients over a 4- to 5-year period. A study of this magnitude cannot be conducted within a single country. Through Canadian Institutes of Health Research (CIHR) funding, we have initiated enrollment in 20 Canadian ICUs (http://balance.ccctg.ca). The BALANCE research program has also garnered enthusiastic collaboration from critical care trials groups in other countries including the United States, Australia, New Zealand, Saudi Arabia, France, Germany and the United Kingdom, where enrollment and potential parallel funding applications will help to foster accrual of the remaining 40 ICUs.

The adherence to treatment duration protocol was lower than our initial target of 90%, but it was similar to the landmark *PneumoA* study of 8 versus 15 days of treatment for ventilator-associated pneumonia, which detected 18% protocol non-adherence in the 8-day treatment arm [6], and was far lower than the non-adherence rates of as high as 50% seen in some studies of biomarker-guided treatment for infections in critically ill patients [24]. Still, non-adherence to treatment duration protocol, particularly in patients in the 7-day treatment arm, represents the main threat to the validity of the principle BALANCE trial. Non-adherence cannot be completely eliminated because some ICU patients will develop secondary infections or persistent sepsis for which antibiotics will be re-initiated or continued, and it is appropriate for those patients to receive treatment, as would occur outside of a trial. We aim for a pragmatic approach to intervention duration, and for patient care. However, several lessons learned from this pilot RCT will enable us to minimize non-adherence in the larger RCT, including: use of delayed randomization when imaging is pending to rule out abscesses or confirm success of initial source control procedures; daily research coordinator contact with the clinical team to monitor for premature or delayed antibiotic discontinuation; and real-time involvement of both infectious diseases and ICU site co-investigators in discussing cases of potential non-adherence. The fact that adherence rates were higher in the second versus the first half of the enrollments at each site suggests that adherence rates may improve significantly over time as the main RCT unfolds. Nevertheless, in the principle BALANCE RCT it will be important to conduct both a primary intention-to-treat analysis and a secondary per-protocol analysis; the strength of the inference of non-inferiority will depend on whether the results of the two analytic approaches are in harmony.

We have not measured treatment group-separated clinical and safety outcomes: the pilot RCT is inadequately powered to assess these outcomes. Spurious differences between groups could lead to unnecessary concerns about embarking on the main RCT, while similar outcomes between groups could lead to premature acceptance of non-inferiority and the adoption of shorter-duration treatment [23]. Instead, this is an internal pilot and the patients enrolled to date will be included and analyzed within the principle RCT. Nevertheless, from the aggregate clinical outcomes in the cohort of 115 randomized patients we have still been able to confirm low rates of antibiotic-related adverse events, and a wide bi-modal distribution of antibiotic-free days. Importantly, the overall rates of *Clostridium difficile* and secondary infections with antibiotic-resistant organisms are high enough that we may be able to detect potentially important benefits of shorter treatment in the principle RCT. For example, the main trial will have 85% power to detect a reduction in *Clostridium difficile* to 3% from 5% with 7 versus 14 days of antibiotics.

One important limitation of the BALANCE pilot and main RCT is that we will only be able to compare outcomes among patients with two fixed durations of treatment, 7 versus 14 days, rather than giving individual patients the minimum duration necessary for curing their particular infection. However, we believe that there are currently no adequate clinical indicators of cure in critically ill patients with bloodstream infection since host signs of sepsis can persist beyond cure of the triggering infection. Although biomarkers, such as PCT, offer a promising means to individualize and reduce antibiotic treatment durations [25, 26], there has been poor adherence to PCT guidance in key trials [24], and this technology has not yet been widely adopted. The results of the BALANCE RCT could be potentially synergistic with prior and subsequent biomarker-focused trials of treatment duration; if 7 days is non-inferior to 14 days of treatment this may lead to greater physician adherence to biomarker-guided stopping rules in the future. Moreover, the blinded PCT results from our study were above the 0.25-IU/mL treatment threshold in the majority of patients at both day 7 and at day 14, suggesting that PCT-guided treatment has the potential to lead to treatment prolongation, rather than reduction, in this clinical context. Finally, as in other studies that compare two arbitrary and fixed intervention thresholds (such as the TRICC trial of transfusion thresholds) [27], an adequately powered trial will provide a benchmark duration of therapy around which to make individualized treatment decisions.

## Conclusions

In the BALANCE pilot RCT, we have documented that randomization of patients to 7 versus 14 days of treatment is acceptable and feasible. Based on these findings, CIHR has funded the BALANCE main RCT to be conducted in both Canadian and international ICUs, with parallel funding applications in progress or under review in collaborating critical care trials groups in other countries. The BALANCE main RCT will provide foundational evidence regarding treatment duration for our sickest and most vulnerable patients. If 7 days is non-inferior to 14 days of treatment, the result could be a paradigm shift in antibiotic treatment durations for these patients, with immediate substantial healthcare savings, and downstream reductions in *Clostridium difficile* and antimicrobial resistant pathogens.

## Additional file

Additional file 1: CONSORT 2010 Checklist of information to include when reporting a pilot or feasibility trial*. (DOC 226 kb)

## Abbreviations

APACHE: Acute Physiology And Chronic Health Evaluation
CCCTG: Canadian Critical Care Trials Group
ICU: Intensive care unit
IQR: Interquartile range
LOS: Lengths of stay
PCT: Procalcitonin
RCT: Randomized clinical trial
